# Provision of low‐aflatoxin local complementary porridge flour reduced urinary aflatoxin biomarker in children aged 6–18 months in rural Tanzania

**DOI:** 10.1111/mcn.13499

**Published:** 2023-03-09

**Authors:** Neema Kassim, Francis Ngure, Laura Smith, Paul C. Turner, Rebecca Stoltzfus, Edna Makule, Nyabasi Makori, Erica Phillips

**Affiliations:** ^1^ Department of Food Biotechnology and Nutritional Sciences, School of Life Science and Bio‐Engineering The Nelson Mandela African Institution of Science and Technology (NM‐AIST) Arusha Tanzania; ^2^ Independent Research Consultant Arusha Tanzania; ^3^ Department of Public & Ecosystem Health Cornell University College of Veterinary Medicine Ithaca New York USA; ^4^ MIAEH, School of Public Health University of Maryland College Park Maryland USA; ^5^ Goshen College Goshen Indiana USA; ^6^ Division of Nutritional Sciences Cornell University Ithaca New York USA

**Keywords:** infants, low‐aflatoxin diet, reduced exposure, urinary AFM1 biomarker

## Abstract

Aflatoxins are toxic secondary metabolites of fungi that colonize staple food crops, such as maize and groundnut, frequently used in complementary feeding. In preparation for a large trial, this pilot study examined if provision of a low‐aflatoxin infant porridge flour made from local maize and groundnuts reduced the prevalence of a urinary aflatoxin biomarker in infants. Thirty‐six infants aged 6–18 months were included from four villages in Kongwa District, Tanzania. The study was conducted over 12 days with a three‐day baseline period and a 10 days where low‐AF porridge flour was provided. Porridge intake of infants was assessed using quantitative 24‐h recalls by mothers. Household food ingredients used in infant porridge preparation and urine samples were collected on Days 1–3 (baseline) and 10–12 (follow‐up). Aflatoxins were measured in household foods, and AFM1 was measured in urine. At baseline and follow‐up, 78% and 97%, respectively, of the infants consumed porridge in the previous 24 h, with a median volume of 220 mL (interquartile range [IQR]: 201, 318) and 460 mL (IQR: 430, 563), respectively (*p* < 0.001). All 47 samples of homemade flour/ingredients were contaminated with AFs (0.3–723 ng/g). The overall prevalence of individuals with detectable urinary AFM1 was reduced by 81%, from 15/36 (42%) at baseline to 3/36 (8%) at follow‐up (*p* = 0.003). Provision of low‐aflatoxin porridge flour was acceptable to caregivers and their infants and successfully reduced the prevalence of detectable urinary AFM1 in infants, thus, confirming its potential to be tested in future large‐scale health outcomes trial.

## Key messages

- Tanzanian infants are commonly exposed to AF through porridge consumption in the complementary feeding period.
- Interventions to test if consumption of low‐AF local foods can reduce prevalence of AF biomarkers are limited.
- Provision of locally processed low‐AF maize: groundnut complementary porridge flour increased porridge consumption and significantly reduced the prevalence of detectable AFM1 by 81%.
- The AFM1 biomarker appeared to be responsive to the intervention.

## Graphical abstract

Tanzanian infants are commonly exposed to AF through porridge consumption in the complementary feeding period. Provision of locally processed low‐AF maize: groundnut complementary porridge flour increased porridge consumption and significantly reduced the prevalence of detectable AFM1 by 81%.

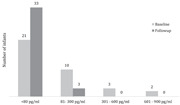

## 1 INTRODUCTION

Aflatoxins (AFs), the secondary metabolites of fungi *Aspergillus flavus* and *A. parasiticus*, are highly toxic members of a larger family of naturally occurring fungal toxins known as mycotoxins. AFs occur in four main forms: aflatoxins B1 (AFB1), aflatoxins B2 (AFB2), aflatoxins G1 (AFG1), and aflatoxins G2 (AFG2). AFB1 is the most prevalent and poisonous, and is classified as a Group I human carcinogen by the International Agency for Research on Cancer (IARC, 1993, 2012). In addition, chronic exposure to AFs is linked to immune system suppression (Jiang et al., 2005; Turner et al., 2003), stunted growth in children (Gong, 2002; Gong et al., 2004; C. Chen, Mitchell, et al., 2018; Shirima et al., 2015; Turner et al., 2007) and micronutrient interference (Watson et al., 2016). Acute high‐level exposure can cause severe liver aflatoxicosis, which can be fatal (Centers for Disease Control and Prevention CDC, 2004; Kamala et al., 2018).

Consumption of AF‐contaminated foods, especially those emanating from susceptible cereals such as maize, oily seeds, and nuts such as groundnuts, is a global public health concern. The global magnitude of exposure to these toxins can vary due to climate, market characteristics, dietary availability and preferences, and food safety control systems. Countries with diversified diets combined with firm regulations that monitor AF levels in susceptible foods are better equipped to protect humans and livestock from ingestion of significant amount of these toxins. However, in countries with limited dietary diversity, widespread production and consumption of susceptible crops, combined with weak enforcement of regulations, ingestion of AF is frequent (Bennett & Klich, 2003).

In Tanzania, maize is widely cultivated and serves as the main staple food to many households (Mtaki, 2020). In addition, it is the main component of complementary food for infants and young children (Muhimbula & Issa‐Zacharia, 2010). Groundnut paste or flour is also commonly used as an ingredient in cereal‐based complementary foods and a condiment in family food, both to provide flavour or in place of cooking oil where the latter is inaccessible. As such, maize and groundnuts are key ingredients of composite flour used to prepare porridge, a traditional complementary food in Tanzania and other parts of the East African region. While maize forms the cereal's major part, groundnut is added as a preblend or during cooking, providing protein, lipids and micronutrients in addition to flavor and taste (Makori et al., 2019).

Maize and groundnut are, however two crops in this region most susceptible to aflatoxin contamination and are the primary source of human exposure (IARC, 2015; Kimanya et al., 2014). In Tanzania, dietary exposure estimates to aflatoxins in rural settings vary from 1 to 786 ng/kg bw/day (median, 12 ng/kg bw/day) (Kimanya et al., 2014), non‐detectable (n.d.) to 120 ng/kg bw/day (Magoha et al., 2016) and n.d. to 24,537 ng/kg bw/day (mean ± SD: 1337 ± 393 ng/kg) (Makori et al., 2019) for infants and young children due to consumption of susceptible staples as part of complementary feeding. A BMDL_10_ of 400 ng/kg bw/day for AFB1 has recently been used as a reference point for the risk characterization of aflatoxins in primary liver cancer; there is as yet no similar estimate for infant growth faltering (EFSA Panel on Contaminants in the Food Chain CONTAM et al., 2020).

The Mycotoxin Mitigation Trial (MMT) study (described by Phillips et al., 2020) delivered a 12‐month dietary intervention using locally produced low‐aflatoxin flours (Ngure et al., n.d.). This pilot study assessed components of the proposed trial intervention, including (a) the effect of distribution of low‐AF flours made from Tanzanian maize and groundnut on infant feeding practices, (b) if these flours were acceptable to caregivers and their infants; and (c) if distribution of such flours lead to significant reductions in detectable urinary aflatoxin M1.

## 2 METHODS

### 2.1 Study site and recruitment of participants

The study was conducted in four villages (Sagara, Machenje, Pandambili A and Mtanana A) of Kongwa district of Dodoma region in Tanzania between April and May 2018. Kongwa district was selected because there is high production and reliance on maize and groundnuts (Mtaki, 2020) for household consumption. The villages were purposively selected based on our results of infant food sampling performed in October and November 2017, that showed differences in cereal intake and detectable and variable levels of AF exposure in this setting. This study built on results from focus groups and recipe trials conducted in the region.

Community health agents collaboratively created a list of all village infants between 6 and 18 months of age. Research staff randomly select eight households per village by pulling names from a hat. The mother/baby dyads were included if the baby had previous consumption of groundnuts with no reported allergy and no illness at recruitment.

### 2.2 Production of low‐AF flours for intervention

The legal limit for processed cereals set by East Africa Community and adopted by Tanzania Bureau of Standards (TBS, 2014a, 2014b) is 10 μg/kg. To produce our low‐AF flours, maize and groundnuts were sourced from Kibaigwa regional market located in Kongwa District. Pre‐screening of maize and groundnuts before purchasing was done by visual inspection for healthy and mould‐free grain. Then three random subsamples of approximately 250 g each were drawn from top, middle and bottom of 100 kg bag and mixed to an aggregate sample for laboratory analysis of AF. Bags with AF at or below 5 µg/kg for maize and 20 µg/kg or below for groundnuts were purchased. Grains were visually sorted, cleaned, and further tested to achieve levels below 5 µg/kg of total AFs (Ngure et al., n.d.).

Grains were milled to obtain porridge flour containing maize and groundnuts at a ratio of 4:1 and sole groundnut flour. Both porridge (1 kg) and groundnut flour (0.5 kg) were supplied to caregivers. Quantities were estimated to last for a week, benchmarked from the quantities reported in the baseline dietary interviews. The proportions were based on the common practice in the study area (Makori et al., 2019; Mollay et al., 2021). Always stainless steel vacuum flask (ALWAYS, China) was also provided for safe and hot storage of babys’ porridge.

### 2.3 Data collection and intervention

This study was designed using the Trials for Improved Practices (TIPS) methodology (Dickin and Griffiths, 1997). The study took place over 12 days, with Days 1–3 being baseline collection, Days 4–12 the intervention period, with follow‐up data collection on Days 10–12. Trained nurses from the local hospital performed all interviews and collected urine samples. Separately trained staff conducted the 24 h recalls and food collection. All interviews were conducted in Swahili. Mother baby dyads were visited on three consecutive days at both baseline and follow‐up (Table 1). On Day 1, mothers were interviewed to understand beliefs and behaviours around infant porridge feeding practices and other types of food consumed by infants. A 24‐h dietary recall was conducted to guide food sampling and record the number of times the baby ate porridge the day before the visit. In this study, three types of maize‐based porridge are commonly fed: (1) thin porridge that can flow off the spoon, (2) thick porridge that stays on the spoon, and (3) stiff porridge or ‘*ugali*’ dishes.

**Table 1:** Schedule of visits for data collection and delivery of the pilot intervention.

Activity	Baseline (days)			6 days interval	Follow‐up (days)		
Data collection/Intervention	1	2	3		10	11	12
Interview about porridge feeding	√						
24 h dietary recall Infant food collection for analysis of AF	√				√		
Provision of vacuum flask, low‐AF porridge^a^ and groundnut flour, and feeding messages			√				
Interview about feeding experiences and counselling				√			
Urine collection	√	√	√		√	√	√

^a^ Sufficient low‐AF flour provided on Day 3 for use until Day 12, checked during mid‐study interview.

For each of the AF‐prone foods reported to be consumed by the infant, a random sample of 500 g of grain/flour from household stores was collected. Where only small quantities (<1 kg) of the grain or flour was available, a sample of 100–250 g was collected. To enhance representative sampling, stocks of 20 kg or less were thoroughly mixed by inverting the storage bag about five times before sampling. Five subsamples of about 100 g each were randomly drawn and mixed to an aggregate sample. In a few households with larger grain stocks (>20 to 100 kg), a sampling spear was used to draw 10 random subsamples of about 100 g each from different parts of the storage bag to obtain an aggregate sample. In both cases, the aggregate sample was mixed thoroughly, and then a subsample of 500 g or less, depending on the stock quantity, was drawn. The samples were tightly packed in dry sealable Ziplock bags and kept at −20°C for AF analysis.

On Day 3 of the baseline study, low‐AF flours were provided. Guidance was provided to the mothers to: (a) use the provided preblended porridge flour when making porridge for the index infant; (b) make a thick porridge, with a maximum ratio of four parts water to one part preblended flour; (c) use porridge made from the provided flour when feeding the index infant, but continue to give nutritious sauces and relishes as normal; (d) use the groundnut flour provided instead of any other groundnut flour when cooking food (even family food) that the index infant would eat; (e) place cooked porridge in the vacuum flask provided to keep it safe and hygienic; and (f) continue breastfeeding as normal.

After the final baseline visit, mothers practiced feeding porridge and other foods made from the provided flours for 1 week. One visit occurred midway through the week to attempt to resolve any challenges cooking and/or feeding the flours. Additional low AF flours were provided at this visit if needed. Daily at baseline and follow‐up visits, infant urine samples were collected using paediatric urine bags and then transferred to sterile urine containers. These were stored frozen (−20°C) for AFM1 analysis.

### 2.4 Analysis of AFs in food samples

Upon collection, samples of grain and flour were stored at −4°C in the transitory sample receipt laboratory in the Kongwa District Hospital, then transported to Nelson Mandela African Institution of Science and Technology (NM‐AIST) laboratory and stored at −20°C before AF analysis. For grains up to 250 g were homogenized in a heavy‐duty RIRIHONG 500A Multifunctional Grinder to a fine flour. Five grams of the flour was mixed with 25 mL of 70% analytical grade methanol (Loba Chemie) for maize or 80% methanol for high matrix effect samples of groundnut, sorghum, rice, millet, finger millet and composite flour. The mixture was extracted on a Stuart Orbital Incubator SI600C (Bibby Scientific Ltd) mechanical shaker at 200 rpm for 20 min. The mixture was allowed to settle and then centrifuged for 5 min at 2465*g* using Eppendorf AG 5810. While low matrix filtrates were ready for the assay, high matrix filtrate required an additional step of dilution (10‐fold) with reconstituted wash buffer solution supplied with the ELISA kit. The diluted high‐ and undiluted low‐matrix filtrates were analysed for total aflatoxins using Helica AF ELISA kit lots AF 120117 and ALM 113017 (Helica), respectively, according to the manufacturer's specification. Enyme‐linked immunosorbent assay (ELISA) plates were read on a Microplate Reader (BIOTEK EL808) at 450 nm. Results of unknown samples were compared with the standard curve to obtain AF concentrations in ng/g. We ensured coefficients of variation of not greater than 5% for duplicates. Samples with CV above 5% were reanalysed. Limit of detection for both kits was 0.05 μg/kg.

### 2.5 Analysis of AFM1 in urine

Urine samples were stored frozen at −20°C in aliquots at the laboratory located in the Kongwa District Hospital, then transported on liquid nitrogen to NM‐AIST laboratory. AFM1 was analysed using the Helica Urinary AFM1 ELISA kits lot AFLM 112917 (Helica) according to the manufacturer's specification (limit of detection of 80 pg/mL) and with modifications described by Smith et al. (2017). Urine samples were thawed at room temperature, and centrifuged using a benchtop micro‐centrifuge (VWR 2400‐37, rotor 2434‐37) at 95*g* for 1 min to pellet solids. The supernatant was diluted with distilled water at a 1:10 dilution, and AFM1 was measured in duplicate according to the manufacturer's instructions. ELISA plates were read on a Microplate Reader (BIOTEK EL808) at 450 nm. Samples measuring above the standard curve were repeated following additional dilutions. An internal sample was measured across each plate to assess inter‐plate variation. Samples were analysed in duplicate and those with a coefficient of variation (CV) of greater than 7% were re‐measured. Urine samples from each individual child/day were analysed separately.

### 2.6 Statistical analysis

R version 4.12 was used for all analyses. Specifically, amounts of porridge consumed before and after the pilot intervention are reported in median with interquartile ranges, and compared using paired sample *T*‐test. Wilcoxon signed‐rank test was used to explain differences between frequency of consumption at baseline and follow‐up visits, and compare prevalence of AFM1 biomarkers before and after the pilot intervention. Spearman's correlation and regression models were used to draw association between age, frequency and volume of porridge consumption.

### 2.7 Ethical consideration

Ethical approval was obtained from Cornell Institutional Review Board (IRB)—USA, and the National Institute for Medical Research (NIMR)‐Tanzania. Administrative permission to visit villages and households was sought from the National through Regional, District and village authorities. Mothers provided informed consent, using a signed written consent form in Swahili to participate in the study.

## 3 RESULTS

**Figure 1:**
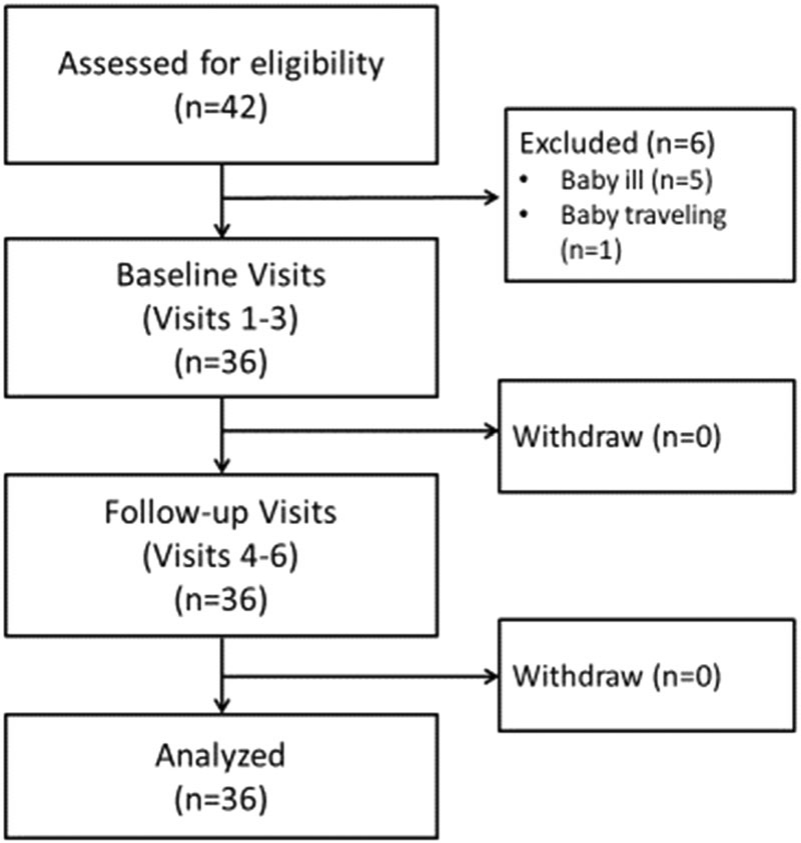
Flow chart of the recruited infants.

Forty‐two infants were assessed for eligibility, of which 36 were recruited; 6 babies were ineligible, due to illness (*n* = 5) and travel (*n* = 1) (Figure 1). Majority of the recruited households were from two main ethnic groups: the Kaguru (50%) and Gogo (33%), with 17% belong to other smaller groups. About 28% of mothers attended but did not complete primary school, 63.9% completed primary school, and only 9% completed secondary school. Babies were between the age of 6–9 (*n* = 12), 10–12 (*n* = 14) and 13–16 (*n* = 10) months, and 52.8% were boys (Table 2). All babies had been introduced to cereal‐based porridge at baseline. The majority, especially from the age of 8 or 9 months and above, were introduced to family foods in addition to porridge.

**Table 2:** Demographic characteristics of respondents.

Characteristics	Number (*n*)	Percentage
Sex of the children		
Boys	19	52.8
Girls	17	47.2
Children age (months)		
6–9	12	33.3
10–12	14	38.9
13–16	10	27.8
Ethnic group		
Kaguru	18	50
Gogo	12	33.3
Others	6	16.7
Mothers’ education		
Below primary education	10	27.8
Primary education	23	63.9
Secondary education	3	8.3

**Figure 2:**
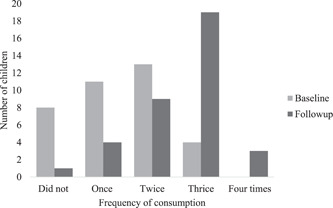
Daily porridge consumption frequency in the past 24 h as reported by mothers at baseline and follow‐up (*n* = 36).

**Figure 3:**
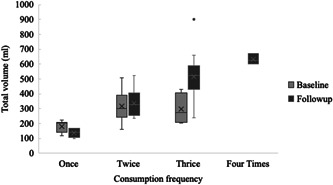
Total daily porridge consumption volume (mL) ranges per day at baseline (*n* = 28) and follow‐up (*n* = 35), grouped by daily consumption frequency.

At baseline, 78% of babies fed on porridge in past 24 h; of these, 39%, 46% and 14% consumed porridge once, twice and thrice a day, respectively. In the follow‐up study, 97% of babies fed on porridge in the past 24 h; of these, 11%, 26%, 54% and 9% consumed porridge once, twice, thrice and four times a day, respectively. While the proportion of babies who did not consume porridge was reduced from 8 (22%) at baseline to 1 (3%) at follow‐up, the shift in porridge consumption frequency between once and four times a day also increased significantly, *p* < 0.001 (Figure 2). The amount of porridge consumed at baseline (median 220 mL: IQR, 201, 318 mL; range 120–509 mL) was significantly lower than that at follow‐up (median 460 mL: IQR, 430, 563 mL; range 100–900 mL (*p* < 0.001). The increase in the volume of consumption appeared to be mostly driven by an increase in the number of daily feedings rather than an increase in any given meal (Figures 2 and 3).

This increase in porridge consumption was also witnessed by mothers during the reinforcement visit, where all mothers stated that they were happy with the provided preblended porridge and groundnut flour and that the infant seemed to like the foods made with these ingredients. Seventy‐eight percent of mothers (28/36) said their infant consumed more porridge when made with the provided flour than when made as usual in the household.

In addition to thin or thick porridge, babies also consumed family foods. At baseline, stiff porridge had the highest frequency: 22.4% (37/165), mango juice (3%) and others (< 3%). In the follow‐up visit, the percentage of stiff porridge consumption was reduced to 1 out of 165 observations (0.6%), with an increased consumption of fresh roasted maize (4.5%), watermelon (3.9%), orange (3.9%) and pumpkin (3.9%).

### 3.1 AF contamination of home‐made porridge ingredients

At baseline and follow‐up, 47 (43 at baseline and 4 at follow‐up) samples were collected, consisting of homemade composite flour or ingredients of porridge flour consumed by infants in the previous 24 h (Table 3). At follow‐up, only four homemade food ingredients were collected because infant foods predominantly ate the porridge made from provided pre‐blend low‐AF flour due to the nature of the intervention. All homemade food ingredients tested had detectable AFs. The overall range for maize was 0.3–25.8 μg/kg (*n* = 33), and for groundnut, 5.0–723 μg/kg (*n* = 8). At baseline groundnuts had a median concentration of 19 times greater than maize (Table 3).

**Table 3:** Aflatoxin contamination of ingredients of home‐made porridge consumed by the infants in the past 24 h.

Sample type (*n*)	AF positive	AF (μg/kg)				
		Range	Mean	Median	>5^a^	>10^b^
At baseline						
Maize (32)	32 (100%)	0.3–25.8	2.6	1.0	2 (6.3%)	1 (3.1%)
Groundnuts (7)	7 (100%)	5.0–723	114.1	19.0	6 (85.7%)	4 (57.1%)
Others (4)	4 (100%)	4.0–6.5	5.5	5.7	3 (75%)	0
At follow‐up						
Maize (1)	1 (100%)	1.2	‐	‐	‐	‐
Groundnuts (1)	1 (100%)	5.2	‐	‐	1 (100%)	‐
Others; rice (2)	2 (100%)	5.2–6.2	5.7	‐	2 (100%)	‐

*Note*: Others at baseline include finger millet at 6.5 μg/kg, composite flour at 4.0 μg/kg, two rice samples at 5.7 and 5.8  μg/kg.

^a^ Limit for AFB1.

^b^ Limit for total AFs in Tanzania.

### 3.2 Prevalence of AFM1 urinary biomarkers

**Figure 4:**
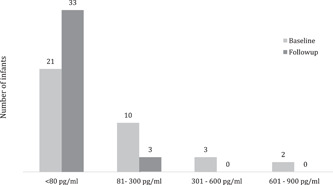
Number of infants (*n* = 36) with detectable AFM1 at baseline (*n* = 15) was significantly (*p* = 0.003) reduced (*n* = 3) at follow‐up. <80 pg/mL is non‐detects.

Three urine samples were collected on consecutive days from each of the 36 infants at both the baseline and the follow‐up visits, resulting in 216 urine samples. Out of the three samples collected from each infant, 42% (15/36) of infants had detectable urinary AFM1 in at least one sample at baseline, which was significantly reduced to 8% (3/36) (*p* = 0.003) at follow‐up. Overall, the intervention reduced the prevalence of detectable urinary AFM1 in infants by 81%. In addition, baseline samples that were detectable (AFM1 range 80–707 pg/mL) tended to be higher than those following the intervention (range 83–143 pg/mL), and notably 6/36 at baseline exceeding 150 pg/mL of AFM1 versus none in the follow‐up (Figure 4).

## 4 DISCUSSION

This study tested if low‐AF porridge flours made from local maize and groundnut altered feeding behaviour was acceptable to caregivers and their infants and how their distribution affected the urinary aflatoxin M1 biomarker in infants in preparation for a larger trial. In addition, typical infant‐fed foods in this region were tested for AF.

The intervention increased the percent of infants who consumed cereal‐based porridge from 78% at baseline to 97%. Daily volume and frequency of porridge consumption was also higher the follow‐up visits. This increase in porridge consumption was expected due to the provision of flour, the message of replacing stiff porridge with thin porridge made from the provided low‐AF flour, and the provision of the vacuum flask for hot storage of thin/thick porridge that encouraged mothers to prepare enough porridge to feed babies as needed all day long. In addition, porridge made from the provided flour appeared preferred over those made from household flour, as mothers reported that their infant consumed more porridge per day when made with the provided flour than when made as usual in the household.

The increase in the volume of consumption appeared to be mostly driven by an increase in the number of daily feedings rather than an increase in any given meal. While overall volume consumed and frequency of porridge consumption were positively associated, the two were not influenced by the age of the child, though statistical power is limited in this pilot. This implies that the frequency and amount of porridge consumed by a child in this study may be determined by other factors such as accessibility and preference. At greater than 6 months, children are also introduced to family food in addition to complementary feeding; this might have decelerated porridge consumption at study entry.

All household samples of flour or ingredients of flour used in complementary feeding were contaminated with aflatoxins. Maize and groundnuts, which account for 68% and 14% of all tested samples, respectively, were each contaminated across a two log range, groundnuts more frequently exceeding the 10 μg/kg regulation (TBS, 2014a, 2014b). These findings are limited in number but compare similarly with other studies (Bennett & Klich, 2003; Kimanya et al., 2014; Magoha et al., 2016). The levels of AF in some samples further supports a concern for infant exposure to AFs early in life as complementary foods are introduced in many parts of the East African region (Muhimbula & Issa‐Zacharia, 2010, Makori et al., 2019).

Typical aflatoxin exposure can be assessed in high‐risk populations using aflatoxin–albumin biomarkers (Turner and Snyder, 2021); however, their use in short‐term interventions is restricted due to the long half‐life of this marker. By comparison, urinary AFM1 better captures exposure changes in studies over several days/weeks. Due to the more transient nature, urinary AFM1, to some extent, reflects a combination of AF levels in food, quantities of food consumed, and a component possibly more relevant for infants, the distribution or timing of feeding activities. Thus, this study actively sought to capture 3‐day measures to better assess the efficacy of the intervention. The LOD of detection in our assay was higher than some other studies (G. Chen, Gong, et al., 2018; Ezekiel et al., 2022; Polychronaki et al., 2008), and while all sampled food at baseline was found to be contaminated, some infants that were exposed would not necessarily be detected by the urinary AFM1 measure in this study. However, the study was able to quantify those with moderate to high level of urinary AFM1, arguably those of most public health concern. In our study, urinary AFM1 was detected frequently in infants at baseline, with several samples exceeding 150 pg/mL. Following several days of using low‐AF flours (<5 μg/kg), this intervention reduced both the frequency of detects and lowered the range observed of those detected at follow‐up, indicating significant efficacy in reducing the burden of exposure.

With a small study of this nature care is needed in attempts to compare these urinary data with other studies. Nevertheless, urinary AFM1 levels in our study (range of detects 80–707 pg/mL) are clearly of some concern. For example, urinary AFM1 measured in regions regarded as high risk, for example, Guinea (24–48‐month‐old) and Nigeria (3–18‐month‐old, *n* = 42), and of moderate risk Egypt (12–30=month‐old, *n* = 50), respectively, had urinary AFM1 detected in samples with 32/50 detected (range 8–801 pg/mL), 5/42 detected (32–505 pg/mL) and 4/50 (range 5–6 pg/mL), respectively (Ezekiel et al., 2022; Polychronaki et al., 2008). A recent survey of infants (*n* = 84) aged 6–16 months from three Tanzanian villages (G. Chen, Gong, et al., 2018) found roughly a similar pattern of AFM1 concentrations, though levels varied significantly by village; Nyabula (Iringa region) non‐detect to 281 pg/mL, Kikelelwa (Kilimanjaro region) non‐detect to 30 pg/mL, and Kigwa (Tabora region) 15–1950 pg/mL. These authors demonstrated a statistically significant albeit modest (*r* = 0.49, *p* < 0.001) relationship between urinary AFM1 and the aflatoxin–albumin biomarker; the latter has been used in studies to assess growth faltering (C. Chen, Mitchell, et al., 2018; Gong et al., 2004; Shirima et al., 2015; Turner et al., 2007). Extrapolation of that association would suggest that infants in our study at baseline with levels of AFM1 above 150 pg/mL may overlap with exposures (based on AF–albumin data) of concern for infant growth. However, given the modest correlation and wide confidence intervals between the two markers reported by G. Chen, Gong, et al. (2018), such a comparison with our data needs to be viewed with caution.

## 5 CONCLUSION

This study population had high frequency and quantity of consumption of porridge made with homemade AF‐contaminated ingredients. Our pilot study found high acceptability and low barriers to recommended feeding practices using the provided low‐AF flours. Building from these results, the MMT trial intervention distributed 4:1 maize to groundnut porridge flour and separate groundnut flour, using AFM1 in a cohort of infants to measure AF exposure levels and adherence to intervention.

## AUTHOR CONTRIBUTIONS

Neema Kassim designed and implemented the study, performed data analysis and interpretation, developed first draft and revised all versions of the manuscript. Francis Ngure designed the study, developed AF‐low food and performed AF analysis of food. Laura Smith designed the study, guided and conducted AFM1 analysis, interpreted data and critically reviewed the final manuscript. Paul C. Turner guided AFM1 analysis, interpreted data and critically reviewed all versions of the manuscripts. Rebecca Stoltzfus  designed the study, interpreted data and critically reviewed all versions of the manuscripts. Edna Makule  implemented the study. Nyabasi Makori implemented the study. Erica Phillips designed and implemented the study, interpreted data and critically reviewed all versions of the manuscripts. All authors have read and approved the final manuscript.

## CONFLICT OF INTEREST STATEMENT

The authors declare no conflict of interest.

## Data Availability

Data available on request from the authors.
